# Use and Effectiveness of Electrosuit in Neurological Disorders: A Systematic Review with Clinical Implications

**DOI:** 10.3390/bioengineering10060680

**Published:** 2023-06-02

**Authors:** David Perpetuini, Emanuele Francesco Russo, Daniela Cardone, Roberta Palmieri, Andrea De Giacomo, Raffaello Pellegrino, Arcangelo Merla, Rocco Salvatore Calabrò, Serena Filoni

**Affiliations:** 1 Department of Engineering and Geology, University G. D’Annunzio of Chieti-Pescara, 65127 Pescara, Italy; david.perpetuini@unich.it (D.P.); d.cardone@unich.it (D.C.); arcangelo.merla@unich.it (A.M.); 2 Padre Pio Foundation and Rehabilitation Centers, 71013 San Giovanni Rotondo, Italy; emanuele.russo@centripadrepio.it (E.F.R.); s.filoni@centripadrepio.it (S.F.); 3 Translational Biomedicine and Neuroscience Department (DiBraiN), University of Bari “Aldo Moro”, 70124 Bari, Italy; r.palmieri20@studenti.uniba.it (R.P.); andrea.degiacomo@uniba.it (A.D.G.); 4 Department of Scientific Research, Campus Ludes, Off-Campus Semmelweis University, 6912 Lugano, Switzerland; raffaello.pellegrino@uniludes.ch; 5 IRCCS Centro Neurolesi “Bonino-Pulejo”, 98124 Messina, Italy

**Keywords:** transcutaneous electrical nerve stimulation (TENS), spasticity, cerebral palsy, stroke, rehabilitation, electro suit, neuroplasticity

## Abstract

Electrical stimulation through surface electrodes is a non-invasive therapeutic technique used to improve voluntary motor control and reduce pain and spasticity in patients with central nervous system injuries. The Exopulse Mollii Suit (EMS) is a non-invasive full-body suit with integrated electrodes designed for self-administered electrical stimulation to reduce spasticity and promote flexibility. The EMS has been evaluated in several clinical trials with positive findings, indicating its potential in rehabilitation. This review investigates the effectiveness of the EMS for rehabilitation and its acceptability by patients. The literature was collected through several databases following the Preferred Reporting Items for Systematic Reviews and Meta-analyses (PRISMA) statement. Positive effects of the garment on improving motor functions and reducing spasticity have been shown to be related to the duration of the administration period and to the dosage of the treatment, which, in turn, depend on the individual’s condition and the treatment goals. Moreover, patients reported wellbeing during stimulation and a muscle-relaxing effect on the affected limb. Although additional research is required to determine the efficacy of this device, the reviewed literature highlights the EMS potential to improve the motor capabilities of neurological patients in clinical practice.

## 1. Introduction

Transcutaneous electrical nerve stimulation (TENS) is a non-invasive treatment technique administered through surface electrodes and able to promote voluntary motor control in patients with central nervous system injuries [1]. TENS is frequently utilised for the purpose of managing pain [1], but its applications in rehabilitation extend beyond pain relief. In fact, TENS has the potential to modulate nerve and muscle activity, thereby facilitating improvements in muscle strength, spasticity reduction, motor control enhancement, and overall rehabilitation support [2]. Particularly, TENS stimulates sensory nerves in the skin, sending non-painful electrical impulses that compete with pain signals, effectively reducing the perception of pain, in accordance with the Pain Gate Control Theory [3]. In addition, the application of electrical stimulation elicits the activation of sensory nerves, which, in turn, triggers the release of endorphins [4]. This mechanism provides analgesic effects and fosters a feeling of overall wellness. TENS has also the capacity to modulate the excitability of nerves, thereby influencing their signal transmission capabilities. Specifically, nerve excitability can be modulated by TENS, based on the electrical stimulation parameters employed. Specifically, it is known that low-frequency TENS has inhibitory effects on nerve transmission, whereas high-frequency TENS is believed to have facilitatory effects on nerve conduction [1]. Furthermore, TENS has the capability to elicit muscle contraction by directly stimulating muscle fibres. This effect permits to enhance muscle strength, facilitate re-education, and ameliorate motor control. The tool increases muscular strength and passive joint range of motion, reducing pain and spasticity [5]. The effectiveness of electrical stimulation to improve outcomes across the International Classification of Functioning (ICF) domains [6] of body structure and function (e.g., clinical Modified Ashworth Scale, MAS), as well as activity (e.g., walking and moving), has been supported by several systematic reviews carried out in the last decade [7,8,9]. This is especially true when used in addition to conventional therapy such as physiotherapy and botulinum toxin injection. However, it is worth highlighting that there are not any specific recommendations for stimulation parameters and dosage to optimise the benefits of TENS [10,11]. While low frequencies have been used and have shown proof in reducing spasticity, the most typical stimulation regimen, for example, uses a high-frequency setting of about 100 Hz [11,12].

The Mollii electrosuit (EMS) (Exoneural Network AB, Danderyd, Sweden) is a full-body garment with built-in electrodes that deliver electrical stimulation to the muscles, with the goal of reducing spasticity and increasing flexibility and range of motion. Integrating TENS into a wearable form, EMS offers several advantages for rehabilitation purposes. In fact, the electrodes placed on the body garment of the suit allow precise targeting of specific muscle groups. This enables customised therapy tailored to the individual’s specific needs and areas of impairment. Moreover, the wearable features of EMS enable users to engage in therapy while performing functional activities and movements. This promotes a more natural and integrated approach to rehabilitation, allowing individuals to practice activities of daily living and functional tasks, and enhancing the potential for consistent and frequent use, which is essential for optimal therapeutic outcomes. The EMS was developed by a Swedish company called Inerventions AB, (Danderyd, Stockholm) founded by Fredrik Lundqvist, MD, who was inspired to develop the suit after seeing the positive effects of electrical stimulation on individuals with spasticity and other neurological conditions. The development of the EMS took several years and involved collaboration with a team of engineers and designers. The goal was to create a wearable device that was comfortable and easy to use, providing also effective electrical stimulation to the muscles. The first prototype of the EMS was developed in 2010 and subsequent iterations were developed and tested over the next several years.

**Figure 1:**
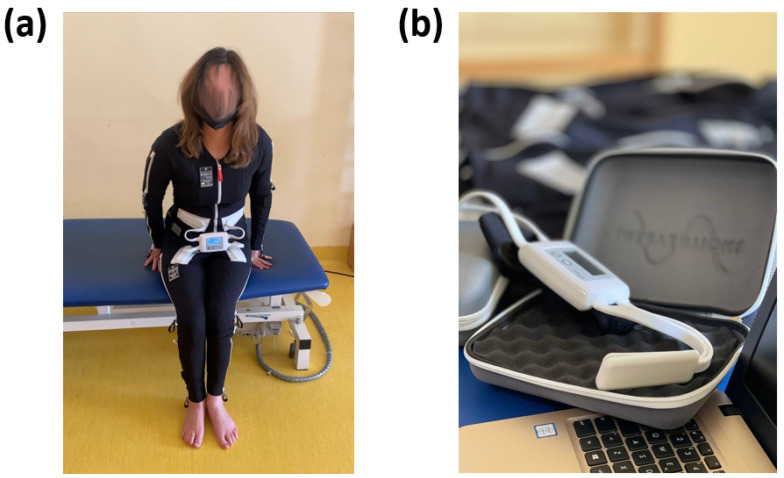
(**a**) wearable garment with 58 electrodes embedded, (**b**) control unit able to modify and configure the parameters of the EMS.

The essential constituents of the EMS encompass a bodily attire (Figure 1a) and a regulating apparatus (Figure 1b).

The wearable apparel is composed of pliable and ventilated material and incorporates 58 distinct electrodes that are tactically positioned across significant muscular regions. The handheld control unit is utilised to modify the device’s configurations and program it in accordance with the user’s requirements. The interface of the control unit is designed to be user-friendly, enabling the user to navigate through the menu options and choose from a range of settings such as intensity, duration, frequency, and pattern of stimulation. It is noteworthy that the control unit generally possesses the capacity to retain numerous programs that are specific to individual users. This feature facilitates convenient accessibility to diverse configurations for distinct users or separate sessions. The retrieval of stored programs obviates the necessity of reprogramming on a recurring basis. Furthermore, certain iterations of the EMS exhibit wireless communication between the control unit and the body garment. The wireless connectivity facilitates the transmission of pre-programmed configurations from the control unit to the electrodes in a live setting. Furthermore, it allows the therapist or user to oversee and modify the stimulation parameters throughout the session. The power source for the control unit is contingent upon the specific model with options including either a rechargeable battery or replaceable batteries. The device has been engineered with safety mechanisms, including pre-programmed safety thresholds, to avert the possibility of hazardous or excessive stimulation. Furthermore, the control unit may be equipped with an emergency stop button that can promptly terminate the electrical stimulation in case of an exigency.

The electrodes utilised in the EMS modality are of paramount importance in the administration of electrical stimulation to the specific musculature being targeted. The electrodes are positioned on the body garment and establish direct contact with the user’s skin. The electrodes are composed of a conductive substance, typically a specialised textile or silicone, that facilitates optimal electrical conductivity and enhances the efficacy of electrical signal propagation from the control module to the muscular system. The placement of electrodes is determined according to an individual’s assessment and positioned over particular muscle groups on the body garment. The control module transmits electrical signals to the electrodes via wired or wireless means, in accordance with the preconfigured parameters. Upon delivery of electrical impulses to the electrodes, a current is generated through the conductive material, subsequently stimulating the underlying musculature. The application of this electrical current induces controlled muscle fibre contraction and relaxation. The electrodes’ electrical stimulation emulates the inherent signals transmitted by the nervous system to initiate muscle activation. The electrodes facilitate the circumvention or augmentation of the compromised neurological pathways by externally stimulating the muscles, thereby enhancing the efficacy of muscle contraction. The customisation of electrode placement allows the precise targeting of particular muscle groups or individual muscles within a group. The technique of selective stimulation facilitates accurate localisation of the impacted regions and can be customised in accordance with the specific requirements and objectives of the therapy. The design of the electrodes is intended to provide a comfortable and non-irritating experience to the user’s skin. Generally, they are integrated into the textile of the clothing item, guaranteeing a steadfast and consistent positioning while in motion. The electrode material has been engineered to possess hypoallergenic properties and to ensure long-term safety during usage.

The suit underwent clinical trials in Sweden and other countries, and the results showed that it was effective in reducing spasticity, improving motor function, and increasing the quality of life for individuals with neurological conditions fostering the adoption of this technology in clinical practice [13]. However, it should be highlighted that the long-term effects of the EMS should be still investigated, hence the EMS should be considered a tool for the management of spasticity during rehabilitative physiotherapy with short-term benefits. Notably, spasticity is meant a motor dysfunction that is distinguished by involuntary muscle contractions or spasms. The primary characteristic of spasticity is an elevation in muscle tone or rigidity. The elevated tone has the potential to induce muscular rigidity, tautness, and hinderance to passive mobilisation. The muscles that are impacted may demonstrate hyperactive reflexes, leading to uncontrolled contractions or convulsions. Spasticity arises from an interruption in the typical equilibrium of signals between the musculature and the central nervous system. Typically, the musculature is subject to neurological modulation via signals emanating from the central nervous system, specifically the brain and spinal cord, which govern the contraction and relaxation of said musculature. Spasticity is characterised by a disturbance in the communication between the neural pathways that promote muscle contraction and those that promote relaxation, resulting in an imbalance between these signals. Nonetheless, spasticity in the chronic phase is complicated by joint fibrosis and tendon retraction, and the suit cannot work on this. It could be interesting to investigate whether the early and long-term use of this tool may help reduce these orthopaedic complications.

Today, the EMS is used by individuals with a variety of neurological disorders including stroke, multiple sclerosis, brain injury, and cerebral palsy (CP). Particularly, the EMS is employed to reduce spasticity based on the idea of reciprocal inhibition brought on by low-frequency and low-intensity stimulation of a spastic muscle’s antagonist. In fact, stimulation is thought to diminish spasticity in muscles by activating inhibitory interneurons in the spinal cord [14,15]. In addition, the EMS could entail neuroplastic alterations in the brain or spinal cord circuitry. Specifically, spasticity reduction is supposed to be mediated by the activation of large-diameter sensory nerve afferents modulating abnormal interneuron activities in multiple spinal segments by insensitivity to prolonged central excitation due to continuous somatosensory stimulation, accompanied by lower corticomotor neuron excitability, or by synaptic reorganisation of the sensory and motor cortices [12].

Then, the aim of this review is to investigate the use of the EMS for rehabilitation purposes, mainly focusing on CP and Stroke treatment. In addition, challenges for future research and development to assure its effective use in clinical practice are discussed.

## 2. Materials and Methods

This systematic review has been performed in accordance with Preferred Reporting Items for Systematic Reviews and Meta-analyses (PRISMA) statement [16]. Since the work sought to highlight the impact of EMS technology on rehabilitation outcomes (with regard to spasticity), the following research questions (RQ) arose:

RQ1. What are the outcomes of the studies investigating the effectiveness of the EMS for rehabilitative purposes?

RQ2. How is the use of the EMS perceived by the patients?

PubMed/MEDLINE, Web of Science, and Scopus databases were systematically searched from the inception to February 2023.

The search concentrated on the words “Mollii” and “neurological disorders” and/or “stroke” and/or “PCI/cerebral palsy” and/or “rehabilitation” and set up by searching for those words within the following fields: article title, abstract, and keywords. After the duplicates were eliminated, the titles and abstracts of all potentially pertinent papers were independently reviewed by two reviewers (DP and EFR) for inclusion. In case of disagreement, the judgment of a third reviewer led to a consensus. The same two reviewers then retrieved full-text studies and separately checked them for inclusion. If an agreement could not be reached through conversation, a third reviewer’s judgment was used to settle disputes (RSC).

Particularly, case series, observational analytic studies, cross-over, and randomised controlled trials (RCTs) were incorporated in the study. Papers written in a language other than English were excluded.

The following information was taken out: Title, Authors, Year of Publication, Study Design, Participants, Intervention Characteristics, Outcomes, and Main Findings. The systematic review has been registered on PROSPERO database: CRD42023425309.

## 3. Results

This section may be divided by subheadings. It should provide a concise and precise description of the experimental results, their interpretation, as well as the experimental conclusions that can be drawn.

All the included papers were summarised, explaining the data extraction process as well as study features. Given the substantial clinical heterogeneity in terms of study design, intervention, and outcomes measured, a meta-analysis was not carried out.

**Figure 2:**
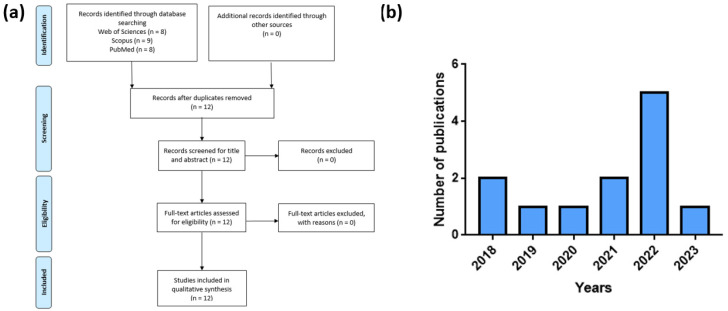
(**a**) Flowchart of the literature screening procedure and number of selected papers, (**b**) number of publications selected per year.

Figure 2a reports the papers selection procedure, whereas Figure 2b reports the number of publications selected per year.

**Figure 3:**
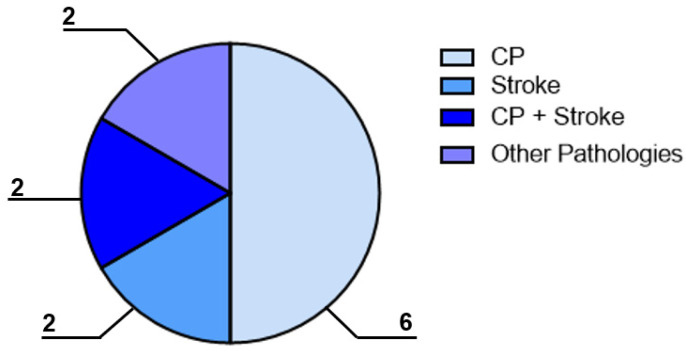
Aerogram reporting the pathologies investigated in the 12 papers included in the review. The numbers of papers selected for each pathology are also shown in the figure.

To answer the first RQ, the categorisation studies concerned the pathology described. Specifically, six of the included papers reported the employment of the EMS for the CP treatment, two papers described the use of the device for the stroke treatment, two papers had a mixed study sample composed of both CP and stroke patients, and two papers investigated patients with fibromyalgia or other pathologies (e.g., Parkinson’s disease), as shown in Figure 3. The manuscripts selected for this review are reported in Table 1.

Studies investigating the employment of the EMS for CP rehabilitation are reported in Section 3.1, whereas research examining the effectiveness of this technology for stroke treatment are described in Section 3.2, and papers exploring the outcomes associated with the employment of this garment for the treatment of fibromyalgia and other pathologies are described in Section 3.3.

**Table 1:** Papers included in the review after the literature review process. The direction of the arrows reported in the table indicates the direction of the significant difference assessed after the treatment for each metric considered (i.e., from bottom to up direction indicates an increase in the variable after the treatment, from top to bottom direction indicates a decrease in the variable after the treatment).

Author	Pathology	Interventions	Outcomes	Results
Bakaniene et al. [17]	CP	EG: EMS (8 patients) C: conventional therapy (8 patients) D: 9 sessions, 3 sessions a week for 3 weeks. Session lasting 60 min.	GMFM PROM Modified Tardieu Scale TUG	GMFM and mobility ↑ There is no superior efficacy of the Inerventions method compared to conventional physiotherapy.
Nordstrom et al. [18]	CP	EG: CP (N = 6) and their parents GMFCS (I-II) C: NO D: 3 months of treatment. Session lasting 60 min, frequency every day	Interview questions about their experiences with the suit: Impact On Body Impact On Self Impact On Activities Improvements During Trial Period Activity And Participation In Daily Activities	Children and parents saw improvements in children’s physical and mental health after the use of the suit. The results are preliminary.
Flodström et al. [14]	CP	EG: CP children (N = 6) C: NO D: employment of the electro-suit for one hour, every other day for three months.	passive range of motion (ROM) muscle tone pain gross motor function participation Canadian Occupational Performance Measure (COPM)	COPM ↑
Arkkukangas et al. [19]	CP	EG: EMS C: NO D: 60 min, four-week intervention period within two weeks after the initial meeting and adjustment of the suit. The treatment regimen was 1 h every other day (3–4 times per week) in daily activities. Adverse events were recorded during the study period.	TUG LSS Box and Block Test Data on pain Sleep MAS	Little to no effect on the outcomes of spasticity, mobility, sitting, upper limb activity, sleep, and pain.
Hedin et al. [20]	CP	EG: EMS C: NO D: The intervention lasted 60 min, three to four times per week, for 6 months	GMFM PROM Modified Tardieu Scale MAS Pain, sleep bowel function	PROM ↑ MAS ↓ Modified Tardieu Scale ↓
Raffalt et al. [21]	CP	EG: EMS C: NO D: 24 weeks treatment with patient-specific muscle stimulation. Before and after the treatment, the patients completed 4 min treadmill walking while trunk accelerometry was obtained.	Largest Lyapunov exponent Complexity index	Largest Lyapunov exponent ↓ Complexity index ↓
Ertzgaard et al. [22]	CP + Stroke	EG: EMS ACTIV C: EMS non activ D: 18 sessions, 3 sessions a week for 6 weeks. Session lasting 60 min.	GAS 10 m walking test, TUG ARAT WMFT MAS	There were no statistically significant differences between the outcomes of the active and non-active treatment periods. During the active period, the 10 m comfortable gait test, time, and number of steps improved. Both the active and non-active periods for the GAS showed improvement.
Jonasson et al. [23]	CP + Stroke	evaluated user experiences with semi-structured interview 6–12 months after previous study	Interest in continuing using the Mollii suit Motivation to use the EMS Usability of the Mollii suit Support in using the Mollii suit.	Responder participants felt hopeful, motivated when experiencing a treatment effect, and disappointed when not.
Palmcrantz et al. [24]	Stroke	EG: EMS C: NO D: 60 min, 21 sessions, 6 weeks	Neuroflexor MAS Fugl-Meyer index grip strength ARAT 10 m walking test 6 min walking test Berg Balance scal Stroke impact scale	FM-UE ↑ FM-LE ↑ ARAT ↑
Pennati et al. [25]	Stroke	EG: EMS (different frequ 20 Hz, 30 Hz) C: PLACEBO D: 60 min, 3 sessions, every day	NeuroFlexor surface EMG MAS passive and active ROM	No significant results
Riachi et al. [26]	Fibromyalgia + Parkinson	EMS therapy for one-hour	VAS just before the intervention (VAS-0) VAS immediately afterwards (VAS-1) VAS after twenty-four hours (VAS-24)	VAS-1 ↓ VAS-24 ↓
Rubio-Zarapiz et al. [27]	Fibromyalgia	60 min session with EMS	Hands temperature Forced expiratory volume in 1 s (FEV1), 6 s (FEV6), and the ratio of both these values (FEV1/FEV6) Pain severity through Numeric Rating Scale (NRS) Muscle oxygen saturation (SmO_2_), total hemoglobin (THb), deoxygenated hemoglobin (HHb), and oxygenated hemoglobin (O_2_Hb) Cortical arousal was measured through the Critical Flicker Fusion Threshold (CFFT) Salivary Biomarkers Functional tests: Chair stand test, Handgrip strength test, 10 m up and go test, one leg balance.	Hands temperature ↓ FEV1 FEV1/FEV6 ↓ FEV6 ↑ NRS ↓ SmO_2,_ O_2_Hb ↑ HHb ↓ Saliva flux ↑ Salivary proteins ↓ Functional tests ↑

To answer the second RQ, the acceptance of the treatment by the patients was investigated. This point could be crucial since the engagement of the patients often influences the positive outcome of the therapy. However, only two papers report about the acceptance of the suit by the patients.

In the Discussion section a summary of the results is presented, deducing from the investigated literature some information regarding the dosage of the EMS and the associated effectiveness. Finally, future challenges in the employment of this technology are mentioned.

### 3.1. The EMS for the CP Treatment

#### 3.1.1. Rationale of the Use of the Tool

Cerebral palsy (CP), a non-progressive neurologic disorder that results from brain damage that happens before cerebral development is complete is the major cause of motor disability in children [28]. It is estimated that the average incidence of CP is 2.08 per 1000 live births, but when only infants weighing less 1500 g are taken into account, the incidence rises of 70 times [29]. Muscle weakness, stiffness, bone abnormalities, and poor balance and coordination are the main features of CP. Moreover, children with CP frequently have low endurance, an increase in the duration of double supports, a decrease in walking speed, as well as impairments in daily activities, community integration, and quality of life [28]. One of the most encompassing and user-friendly systems to rate the seriousness of CP motor abnormalities is the Gross Motor Function Classification System (GMFCS) [30]. This scale rates the child’s independence in fundamental motor skills and divides the patients into five classes, with class 1 being people who can walk on their own and class 5 including people who are unable to move independently [30]. Numerous pharmaceutical strategies [31] and rehabilitative methods have been created [32] to enhance the quality of life and social integration of CP patients. Robotic-assisted gait training (RAGT) is now a popular rehabilitation technique used to help persons with neurological problems improve their gait pattern [33,34,35]. Thanks to its modulatory effects on spasticity, EMS might be a potentially effective tool in the treatment of patients with CP.

#### 3.1.2. Findings of the EMS Employment for the CP Treatment

Recently, researchers investigated the effectiveness of the employment of the EMS to treat the spasticity in CP patients.

Bakaniene et al. [17] studied the effect of the EMS on GMF in 16 children with spastic CP (aged about 5 years) using a randomised controlled trial. The sample was divided evenly between the Inerventions approach (EMS) and the control group. In the Inerventions group, the EMS was applied for one hour per session, three times per week, for three weeks. The duration, frequency, and duration of the functional exercises program for the children in the control group were the same. The GMFM, passive range of motion (PROM), the Modified Tardieu Scale, and the Timed Up and Go test served as outcome measures. While both groups improved in GMFM and mobility, the difference between children treated with the EMS and those treated with conventional therapy was not statistically significant. In addition, neither group experienced a change in PROM or spasticity resulting from the therapies.

Given the limited non-invasive treatment alternatives available at home, Arkkukangas et al. [19] studied the effect of the EMS on CP children aged 4 to 17 years (GMFCS levels 1 to 4) in an effort to determine the suite’s potential use for domestic treatment. The interventions lasted four weeks, and the treatment schedule consisted of one hour three to four times per week. The number of electrodes utilised depends on the spasticity symptoms and muscles requiring inhibition of the participants, and parameters for the stimulation included pulse duration (0–30 ms), frequency 20 Hz, and voltage 20 V. The primary outcome (spasticity) was examined visually using graphed data, and median values were examined for secondary outcomes (mobility, sitting, upper limb activity, sleep, pain, and adherence to treatment). The modified Ashworth Scale was used to assess spasticity (MAS), the Timed Up and Go test (TUG), the Level of Sitting Scale (LSS), and the Box and Block Test were utilised to assess mobility, sitting, and upper limb activity. In none of the seven single case studies, the electro-dress treatment showed little to no effect on the outcomes of spasticity, mobility, sitting, upper limb activity, sleep, and pain.

Flodström et al. [14] evaluate the potential impact of EMS on body function, physical activity, and involvement in self-selected activities. Six children wore the garment for one hour every day for three months. The effect was examined by evaluating PROM, muscle tone, pain, gross motor function, and participation. The total score on the Canadian Occupational Performance Measure (COPM) increased for all individuals, and three of them had significant clinical improvements. Pain was reduced in children who reported pain at the beginning of the research.

Hedin et al. [20] used the EMS on 16 children (8F) with CP for six months (GMFCS I-V age 2–16 y, median age 6.3 y). The intervention lasted 60 min, three to four times per week, and involved the reciprocal inhibition of the spastic muscle. After one, three, and six months of treatment, a significant number of improved muscles were observed for PROM (*p* = 0.000, *p* = 0.001, *p* = 0.014, respectively). Using the MAS, the degree of spasticity decreased considerably at one (*p* = 0.007) and six months (*p* = 0.011). The modified Tardieu reduced considerably after one month (*p* = 0.030), but not after three or six months (*p* = 0.392 and *p* = 0.426, respectively).

Raffalt et al. [21] examined the effect of systematic therapy with the EMS on the nonlinear dynamics and variability of trunk accelerations during walking in unilateral CP children. Twelve patients (mean age: 12 years, range: 7–17 years) with unilateral CP (GMFCS levels 1 and 2) were treated for 24 weeks with patient-specific muscle stimulation using an EMS. Before and after therapy, patients walked for four minutes on a treadmill while trunk accelerometery measurements were taken. The nonlinear dynamics was defined by the largest Lyapunov exponent and complexity index from the multiscale entropy, whereas the movement variability was quantified by the root mean square ratio. Using a paired Student’s *t*-test, pre- and post-treatment differences were analysed Post-treatment, the highest Lyapunov exponent (*p*-value = 0.041) and the complexity index (*p*-value = 0.030) of the anterior–posterior acceleration were dramatically reduced. The study suggests that 24 weeks of treatment with the EMS modifies the nonlinear dynamics but not the variability of trunk accelerations while walking in children with unilateral CP.

### 3.2. The EMS for the Stroke Treatment

#### 3.2.1. Rationale of the Use of the Tool

Stroke is a prevalent, dangerous, and incapacitating health issue on a global scale, and rehabilitation is a crucial aspect of patient care. The estimated global cost of stroke is over $721 billion [36]. Stroke remains the second-leading cause of death and the third-leading cause of death and disability combined (as expressed by disability-adjusted life years lost—DALYs) in the world [36]. In fact, despite a decline in stroke mortality, the prevalence of people living with the effects of stroke has increased due to the aging and growing population. Rehabilitative services are in greater demand as the number of stroke survivors rises [37]. Specifically, it is evident that a brain injury, such as a stroke, causes an imbalance of inhibitory and excitatory impulses that results in upper motor neuron symptoms, and that the location and extent of the lesions result in varying symptoms and degrees of spastic severity [38]. The onset of spasticity after a stroke is highly variable and may occur within or beyond one year. The current understanding of the onset of spasticity is complicated by the role of contractures, which have been assumed to result from spasticity but may play a role in its ethiology [38]. Other potential risk factors for post-stroke spasticity include early arm and leg weakness, left-sided weakness, early reduction in activities of daily living, and smoking history [39]. Robotics and constraint-induced movement therapy are potential treatments for arm’s motor rehabilitation [40]. Exercise, high-intensity therapy, and repetitive task training are promising strategies that may help to improve aspects of gait [41]. EMS could be a valuable and easy-to-use tool to reduce spasticity and improve motor function in such patient populations, thanks to its potential effect on reciprocal inhibition at the spinal cord level.

#### 3.2.2. Findings of the EMS Employment for the Stroke Treatment

Ertzgaard et al. [22] examined the efficacy of the EMS for self-treatment of spasticity in stroke or CP patients with spasticity. The research was a randomised, controlled, double-blind, and cross-over study. Treatments were administered at home, while all follow-ups took place in the two rehabilitation clinics. Twenty-seven participants successfully completed the study (15 strokes and 12 CP). Six weeks were spent using the EMS with and without electrical stimulation (active/non-active period), followed by six weeks without treatment. Changes in mobility, arm-hand ability, spasticity, and pain were measured at baseline and after 6, 12, and 18 weeks using Goal Attainment Scaling (GAS). There were no statistically significant differences between the outcomes of the active and non-active treatment periods. During the active period, the 10 m comfortable gait test, time, and a number of steps improved. Both the active and non-active periods for the GAS showed improvement. However, it should be noted that only 15 of the 27 participants complied with the treatment protocol’s recommended use, and deviations from the protocol were common; consequently, the results should be interpreted with caution.

Palmcrantz et al. [24] investigated the possibility of stroke patients using the EMS at home to treat spasticity. Specifically, 20 stroke survivors with spasticity and hemiplegia used the EMS for six weeks. Through weekly telephone interviews, usability and perceived effects were monitored. The outcome was evaluated using the NeuroFlexorTM method for quantifying the neural component (NC) of resistance to passive stretch (spasticity), the modified Ashworth scale (MAS) for total resistance, Fugl-Meyer, and the Fugl-Meyer index. Motor recovery evaluation for sensorimotor function in the upper (FM-UE) and lower extremities (FM-LE), activity performance with the Action Research Arm Test (ARAT), Berg balance scale, 10 m and 6 min walk tests, and perceived functioning with the Stroke Impact Scale. High compliance was observed in the study (mean 19.25 of 21 sessions). After the intervention, the NC decreased significantly in the wrist flexors of the affected hand (*p* = 0.023), and the FM-UE and FM-LE scores improved significantly (*p* = 0.000 and 0.003, respectively). Except for the ARAT, no significant differences were found between MAS and assessed activity performance. Changes in FM-UE score were significantly and moderately correlated with the perceived effect in the upper extremity (r = 0.498; *p* = 0.025) and with the FM-LE and perceived effect in the lower extremity (r = 0.469; *p* = 0.037).

Pennati et al. [25] investigated the efficacy of one session of stimulation with the EMS at various stimulation frequencies on objective signs of spasticity and clinical measures, as well as the subjective perceptions of the intervention, in twenty patients in the chronic phase after stroke. Every other day, the EMS was used to deliver electrical stimulation for 60 min at two active frequencies (20 and 30 Hz) and in OFF settings (placebo). Using the NeuroFlexor hand and foot modules, spasticity was evaluated with controlled-velocity passive muscle stretches. Complementary tests included surface electromyography (EMG) for characterising flexor carpi radialis, medial gastrocnemius, and soleus muscle activation, the Modified Ashworth Scale, and range of motion. At the group level, analyses revealed that stimulation at any frequency had no significant effect on the NeuroFlexor NC and EMG amplitude of the upper or lower extremities (*p* > 0.35). At the individual level, however, the effect was highly variable, with eight patients exhibiting reduced NC (>1 N) in the upper extremity after stimulation at 30 Hz, five after stimulation at 20 Hz, and three after stimulation at OFF settings. All of these patients had severe spasticity at baseline, as measured by an NC > 8 N. After stimulation at any frequency, the MAS ratings for spasticity and range of motion did not change significantly. In the chronic phase after stroke, 60 min of electrical stimulation with the EMS did not consistently reduce spasticity in the upper and lower extremities. The findings suggest that patients with severe spasticity following a stroke require additional research, including repeated stimulation sessions.

### 3.3. The EMS for Fibromyalgia and Other Pathologies

Fibromyalgia is a disorder characterised by widespread chronic pain, sleep disturbances, physical tiredness, and cognitive issues. Worldwide, fibromyalgia affects all populations, with symptom prevalence ranging from 2% to 4% in the general population [42]. Parkinson’s disease is the second most prevalent neurodegenerative condition, affecting 2–3% of the ≥65-year-old population. Although the clinical diagnosis is performed by evaluating the occurrence of bradykinesia and other cardinal motor symptoms, this pathology provokes also non-motor conditions (e.g., cognitive impairment, autonomic dysfunction, disorders of sleep, depression, and hyposmia) [43].

Riachi et al. [26] investigated the effect of the EMS on pain in adults with various pain diagnoses. In an uncontrolled, open-label study, 200 adults (75 men and 115 women) used EMS therapy for one hour. Seventy-two were diagnosed with Fibromyalgia, 29 with Parkinson, and the frequency of all other diagnoses was 20. Patients were asked to complete a Visual Analogue Scale (VAS) immediately before (VAS-0), immediately after (VAS-1) and twenty-four hours later (VAS-24) the intervention. VAS-0 was 6.5 ± 1.24. A highly significant drop was noted in VAS-1 (3.46 ± 1.4) and VAS-24, (4.72 ± 1.68), paired test *p*-values < 0.001. Using a mixed-effect model to assess VAS change while controlling for gender, age, and type of diagnosis, VAS-1 and VAS-24 decreased significantly. The VAS-1 coefficient was −3036 (*p* = 0.001), and the VAS-24 coefficient was −1789 (*p* < 0.001). There was no influence in results by patients’ diagnoses, age, or sex.

This result was confirmed by Rubio-Zarapuz et al. [27] who investigated the effects of a 60 min session with the EMS on a female fibromyalgia patient in a case report study. The single session with the EMS has been shown to improve pain perception, muscle oxygenation, parasympathetic modulation, and function in female fibromyalgia patients after intervention.

### 3.4. The Acceptance of the EMS

The success of therapy is directly proportional to the patient’s engagement in the treatment. The concept of “rehabilitation engagement” includes the patient’s attitude toward therapy, understanding of the necessity of intervention, need for verbal or physical prompts, active participation in therapy activities, and attendance throughout the rehabilitation program [34,44,45]. Increased participation in rehabilitation is associated with decreased levels of illness denial and negative affective states, as well as increased levels of positive affective states. Thus, researchers began to focus on monitoring the psychophysiological status of patients during therapy with the EMS.

Jonasson et al. [23] interviewed fifteen patients who had used the EMS assistive technology to discuss their experiences. Motivation, support, usability, and outcome are four dimensions recognised by clinicians as essential to the success of technology-based interventions. Positive outcomes described by participants included increased mobility, decreased spasticity, and a reduction in the use of medication for spasticity-related symptoms, although not everyone experienced these benefits. The interest in continuing to use the treatment for home-based training after the study was due to each individual’s experience with the treatment’s efficacy, (initial) motivation and determination to try a new concept, support from family members or home service personnel to use the treatment and overcome potential obstacles, and the suit’s overall usability. The participants’ interest in using the training concept thus depended on the suit’s effectiveness, their motivation to use the suit, the suit’s usability, and the suit’s support. In fact, this study revealed the significance of effective communication with and prompt response from the AT provider.

In addition, Palmcrantz et al. discovered that 60% of the stroke participants reported positive effects, most commonly related to decreased muscle tone (*n* = 9), improved gait pattern function (*n* = 7), and voluntary movement in the upper extremity (*n* = 6). In accordance with these findings, Pennati et al. discovered that 75% of stroke patients reported an overall sense of wellbeing during stimulation, with 25% of patients describing a muscle-relaxing effect on the affected hand and/or foot at both 20 and 30 Hz.

Nordstrom et al. [18] interviewed six children aged 5 to 10 and their parents to investigate their experience of the use of the EMS. The interviews were evaluated using qualitative content analysis, identifying three main themes: the suit’s effect on the image, changes that make a difference, and dealing with a need for change. Each theme comprised subthemes. After wearing the suit, all children reported some impacts on their body, self, and/or certain activities. Throughout the trial time, the parents also experienced improvements. However, the results are preliminary, and a larger study is required to evaluate whether the suit is effective over the long term and whether it can impact children with spasticity’s activity and participation in daily activities.

Flodström et al. [14] observed that the EMS had a good effect on the children’s activity and engagement in self-selected activities. However, the authors suggest that additional research with a larger number of youngsters over a longer period are required to evaluate the impact and utility over time.

## 4. Discussion

In this review, the effectiveness of the EMS is investigated for neurologic rehabilitation purposes. From the literature investigated, a minimum of six weeks of administration of the technology is necessary to obtain statistically significant results. Notably, Ertzgaard et al. [22] found improvements, although not significant, in motor functions in CP and stroke patients after six weeks of treatment for a total of 18 sessions, whereas Palmcrantz et al. [24] observed a significant improvement in motor functions in stroke patients after six weeks of treatment for a total of 21 sessions. These findings suggest that not only the duration of the administration period could influence the effectiveness of the therapy, but also the dosage of the treatment. From this perspective, it is important to highlight that there are no shared protocols regarding the dosage of the EMS. Particularly, the dosage of EMS treatment may vary depending on the individual’s condition, the severity of the symptoms, and the treatment goals. For this reason, the recommended treatment duration and frequency are typically determined by a healthcare professional and may be adjusted over time based on the individual’s response to treatment. Notably, in the user manual of the EMS is reported that sessions of 60 min are recommended.

From this review, it could be deduced that further investigations should be performed in order to increase the efficacy of the EMS in clinical practice. For instance, it should be investigated whether the employment of the EMS could be optimised for specific neurological conditions. In fact, different neurological diseases can affect the body in different ways and understanding the specific condition can help to tailor the use of the EMS to address the unique needs of the individual. In this perspective, the target muscle groups should be identified to maximise the benefits of the suit for the individual, and the intensity of the electrical stimulation should be adjusted to suit the individual’s needs (e.g., individuals with more severe spasticity may require a higher intensity of stimulation to achieve a positive effect), and the duration and frequency of EMS treatment sessions should be optimised to achieve maximum therapeutic benefit. From this perspective, future research should focus on identifying the most effective treatment protocols for different neurological conditions and varying levels of impairment. This can involve investigating the optimal stimulation parameters, electrode placement strategies, duration and frequency of sessions, and the integration of the suit with other rehabilitation approaches. Moreover, exploring the customization and personalisation of EMS-based therapy can lead to improved outcomes. Future studies should investigate the development of algorithms or techniques to tailor stimulation settings and electrode placement to individual characteristics such as muscle tone, motor deficits, and specific movement patterns.

There should be studies to compare EMS therapy with other treatments for spasticity and mobility impairments, such as physical therapy or medication, in order to evaluate the cost-effectiveness of EMS compared to other treatment options. Importantly, studies should investigate how the EMS can be integrated into existing rehabilitation programs for individuals with neurological conditions in order to maximise the positive effects for the patients. In this perspective, it is worth highlighting that the EMS costs around 6000 €, hence it is crucial to investigate the effectiveness of this device since the adoption of the garment in clinical practice could be expensive for the healthcare system. Furthermore, together with the acceptance of the device by the patients, it should be investigated whether the EMS impacts the quality of life for individuals with spasticity and mobility impairments.

It should be also investigated the possibility to employ the EMS as a long-term treatment option for individuals with chronic neurological conditions, comparing its effectiveness to other long-term therapies. Importantly, conducting well-designed clinical trials and comparative studies can help establish the effectiveness of the EMS in various neurological conditions. These studies should include large sample sizes, control groups, and long-term follow-ups to assess the sustained benefits of using the suit. In fact, longitudinal studies examining the long-term effects of using the EMS can provide insights into its impact on functional outcomes, quality of life, and potential secondary benefits such as pain reduction and spasticity management. Understanding the sustainability of the improvements achieved with the suit can guide treatment planning and optimise long-term care. In addition, further investigating the user experience, satisfaction, and adherence to EMS-based therapy can help identify factors that influence compliance and engagement. This research could foster the development of strategies to enhance user acceptance, motivation, and integration of the suit into daily activities. In fact, it is worth highlighting that, as a matter of health policy, most social and economic expenditures are devoted to acute stroke and CP up to the age of 16. In this perspective, the EMS is highly suitable for the treatment of this kind of patients since it is a device that can be used at home, without visiting a rehabilitation facility, and it does not require special expertise to be worn and utilised. Moreover, it could be also utilised by patients with severe conditions (such as those with GMFM IV and V) for whom passive mobilisation is the only option. However, the long-term impact of using the EMS on overall health outcomes, such as cardiovascular health and mental well-being, should be examined.

Importantly, no contraindications of the employment of the EMS are reported in the investigated studies. However, the interference of this device with some implanted devices (e.g., cardiac pacemakers), and possible negative effects during pregnancy should be investigated.

In this perspective, studies combining the EMS with different techniques able to measure physiological changes associated with the treatment should be fostered. In fact, for instance, the impact of the EMS on muscle activation patterns and muscle strength could be investigated by employing electromyography on the target muscles [46,47], and the effect on the wellbeing and psychophysiological state of the individuals could be investigated by measuring changes in heart rate variability and breathing rate [48,49], and the impact on the cerebral plasticity could be investigated through the electroencephalography [50,51] or functional near infrared spectroscopy [52,53]. However, it is known that peripheral electrical stimulation can promote plasticity in the human brain [54]; however, specific studies focusing on the effect of the EMS-based stimulation on the brain are lacking. Importantly, investigating physiological and neurological changes that occur during and after using the suit can provide valuable insights into its therapeutic effects.

Finally, further exploring the use of EMS in paediatric populations can expand its potential applications. Studying its efficacy, safety, and usability in children with developmental disabilities can provide valuable evidence for its effectiveness in this specific population Although further studies should be performed to test the effectiveness of the EMS for rehabilitative aims in clinical practice, this review indeed demonstrates the potentiality of this device to improve the motor functions of CP, stroke, and other neurological disease patients, highlighting the necessity to promote further scientific investigations to maximise the positive effects of the device for the patients. By promoting further scientific investigations and establishing a robust evidence base, the true potential of EMS in rehabilitation could be indeed explored. This will ultimately optimise EMS application, guide clinical decision-making, and improve the overall outcomes and quality of life for patients. Continued research efforts and collaboration between clinicians and researchers are vital in harnessing the full therapeutic benefits of EMS devices for the benefit of patients.

## 5. Conclusions

The EMS is a full-body garment with built-in electrodes that deliver electrical stimulation to the muscles, with the goal of reducing spasticity and increasing flexibility and range of motion. This review investigated the benefits of the employment of the EMS for rehabilitation purposes for CP, stroke, and other neurological disease patients. Although further studies should be performed to test the effectiveness of this device, the literature reviewed demonstrated the potentiality of the EMS in clinical practice to improve the motor functions of neurological patients.

## Data Availability

Not applicable.

## Abbreviations

Action Research Arm Test (ARAT), Canadian Occupational Performance Measure (COPM), Cerebral palsy (CP), Disability-adjusted life years lost (DALYs), Exopulse Mollii Suit (EMS), Fugl-Meyer lower extremities (FM-LE), Fugl-Meyer Upper Extremities (FM-UE), Goal Attainment Scaling (GAS), Gross Motor Function Classification System (GMFCS), International Classification of Functioning (ICF), Level of Sitting Scale (LSS), modified Ashworth Scale (MAS), Neural component (NC), passive range of motion (PROM), Preferred Reporting Items for Systematic Reviews and Meta-analyses (PRISMA), Research questions (RQ), Robotic-assisted gait training (RAGT), surface electromyography (EMG), Timed Up and Go test (TUG), and transcutaneous electrical nerve stimulation (TENS), Experimental group (EG), Controls (C), Treatment duration (D).
